# Cardiovascular Risk Factor Status in Hospitalized Patients With Type 2 Diabetes in China

**DOI:** 10.3389/fendo.2021.664183

**Published:** 2021-07-22

**Authors:** Xiaoyun Yang, Qian Liu, Yuxin Fan, Li Ding, Ruodan Wang, Gang Hu, Ming Liu

**Affiliations:** ^1^ Department of Endocrinology and Metabolism, Tianjin Medical University General Hospital, Tianjin, China; ^2^ Pennington Biomedical Research Center, Baton Rouge, LA, United States

**Keywords:** type 2 diabetes, control rates, cardiovascular risk factors, complications, prevalence

## Abstract

**Background:**

Controlling blood glucose, blood pressure, and blood lipid is of great importance for patients with type 2 diabetes, not only for cardiovascular disease, but also for other complications. Previous studies mainly focused on the control rate of outpatients, and the results were suboptimal, but few studies aimed at the inpatients.

**Method:**

The present study involved 3,245 hospitalized patients with type 2 diabetes from 2013 to 2017 in the Department of Endocrinology and Metabolism of Tianjin Medical University General Hospital. The percentages of inpatients who attained the goals of the China Diabetes Society and the American Diabetes Association were calculated for major cardiovascular risk factors (HbA1c, blood pressure, and blood lipid). The prevalence of microvascular and macrovascular complications was also assessed.

**Result:**

The percentages of patients who met the Chinese Diabetes Society goals—HbA1c <7%, blood pressure <130/80 mmHg, normal lipids, and all three goals—were 26.7, 14.8, 10.4, and 0.2% in 2013 and 30.5, 16.2, 8.0, and 0.9% in 2017, respectively. The percentage of patients who met all three American Diabetes Association goals (HbA1c<7%, blood pressure <140/90 mmHg, low-density lipoprotein cholesterol <2.6 mmol/L) increased from 4.3% in 2013 to 9.0% in 2017. The prevalence of major diabetes complications including coronary heart disease (31.7 *vs*. 31.9%), stroke (16.7 *vs*. 14.8%), diabetic kidney disease (37.9 *vs*. 35.8%), diabetic retinopathy (48.0 *vs*. 46.5%), neuropathy (63.1 *vs*. 61.9%), and diabetic foot (0.8 *vs*. 1.2%) were stable from 2013 to 2017.

**Conclusion:**

During 2013 to 2017, control rates of major cardiovascular risk factors including HbA1c, blood pressure, and low-density lipoprotein cholesterol were improved among hospitalized patients in Tianjin, China.

## Introduction

The prevalence of diabetes has become a major public health issue of the 21st century worldwide. In 2019, a total of 463 million people were estimated to be living with diabetes, representing 9.3% of the global adult population (20–79 years old) (1). This number was expected to increase to 578 million (10.2%) in 2030 and 700 million (10.9%) in 2045. As a result of more sedentary living, consumption of high-energy diets, and other yet-unknown reasons, China became the country with the largest number of diabetes, with 116 million cases in 2019. A recent result from the national survey in mainland China indicated that the overall prevalence of diabetes is 11.2% in 2017, which has a prominent improvement than 2013 (2). The increase of diabetes prevalence places a great burden on the country.

The deleterious health effects of diabetes are largely due to the potentially life-threatening microvascular and macrovascular complications and also due to the comorbidities like hypertension and dyslipidemia (3). Poor glycemic control, dyslipidemia, and hypertension, central obesity, low physical activity, smoking, and older age play great roles in the development of complications of diabetes (4). Thus controlling blood glucose, blood pressure, and blood lipid is of great importance for patients with type 2 diabetes, not only for cardiovascular disease, but also for other complications (5). Several major international and national associations such as the American Diabetes Association (ADA) and the Chinese Diabetes Society have set the targets of treatment and control of major cardiovascular risk factors, such as HbA1c, blood pressure, and blood lipid among patients with type 2 diabetes (6, 7).

However, only a minority of patients reached the targets. A multi-center survey of outpatients with type 2 diabetes from August 2010 to April 2012 in China indicated that the glycemic control rate was only 32.6%, and the triple control rate for glycemia, blood pressure, and lipids was only 11.2% (8). Another investigation from the US showed that control rate of cardiovascular risk factors among US adults was only 24% at goal for all three factors (HbA1c <7%, blood pressure <130/80 mmHg, and low-density lipoprotein (LDL) cholesterol <2.6 mmol/L) in 2009–2010 (9). Meanwhile, a recent meta-analysis, which assessed the achievements of targets according to different guidelines in different countries, reported that the achievements rates were 42.8% for glycemic control, 29% for blood pressure, 49.2% for LDL cholesterol, 58.2% for high-density lipoprotein (HDL) cholesterol, and 61.9% for triglyceride (10). However, most of the previous studies focused on the outpatients, the community diabetes management or the health survey, which mainly includes patients who are at a lower risk of metabolic disorders. It is well established that the characteristics of inpatients are completely different from outpatients and the patients from the health survey. However, very few studies have focused on inpatients especially those hospitalized in the tertiary general hospital, in which the conditions of this group of patients are more comprehensive and serious. Therefore, the aim of our study is to evaluate the metabolic characteristics of the hospitalized patients with type 2 diabetes from 2013 to 2017 in Tianjin, China.

## Methods

### Study Participants

**Figure 1:**
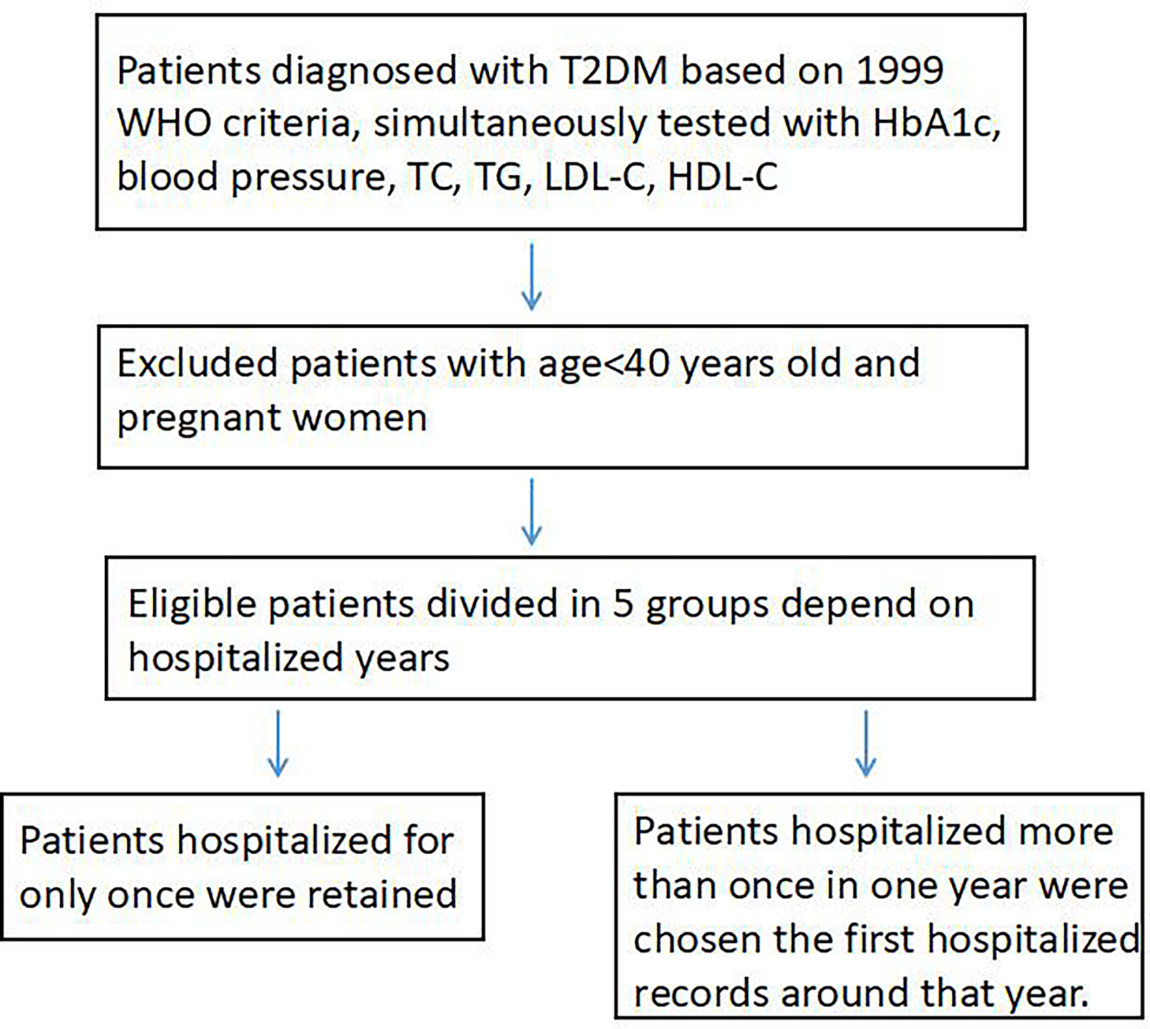
Participant flow chart.

The Tianjin Medical University General Hospital is the largest tertiary comprehensive teaching hospital affiliated to the Tianjin Medical University in Tianjin, China. The Department of Endocrinology and Metabolism is a National Key Discipline (developing) and Tianjin Clinical Key Discipline. Patients are not only local residents, but also residents over the nation. In addition to routine care of patients with endocrine disorders, the department provides comprehensive interdisciplinary treatment for the endocrine patients including diabetes in critical conditions with more complications and/or comorbidities. Most of the hospitalized patients with diabetes in the department have uncontrolled hyperglycemia for a period of time or with at least one of the complications or comorbidities and sometimes experienced acute diabetic complications. The number of hospitalized patients with diabetes is approximately 900 in average per year. We chose type 2 diabetes patients from 2013 to 2017. The inclusion criteria were: (1) adults diagnosed with type 2 diabetes based on the 1999 World Health Organization criteria (WHO) (11) (repeated fasting plasma glucose levels ≥7.0 mmol/L, 2-h plasma glucose ≥11.1 mmol/L during a 75 g glucose oral glucose tolerance test (OGTT), typical symptoms of diabetes with random plasma glucose ≥11.1 mmol/L), or previous diagnosis as type 2 diabetes. When the diabetes classification was difficult to identify, we combined the OGTT results including C-peptide, diabetic autoantibody, and the genetic test for confirmation; (2) adults aged 40 or more excluding pregnant women; (3) simultaneous tests of HbA1c, blood pressure, and blood lipid including total cholesterol, triglyceride, LDL cholesterol, and HDL cholesterol. Patients who were hospitalized for only once per year retained, while patients hospitalized more than once in one year were chosen with the first hospitalized record at that year (**Figure 1**).

### Clinical Measurements and Assessments of Complications

All data were extracted from the medical record and the inpatient electronic medical record system. At admission, trained nurses measured height, weight, and blood pressure using a standard protocol. Body mass index (BMI) was calculated as weight in kilograms divided by height in meters squared. A venous blood specimen was drawn the next morning after hospital admission with at least 12 h fasting. HbA1c was measured using high performance liquid chromatography. Blood lipid profiles including total cholesterol, LDL cholesterol, HDL cholesterol, and triglyceride were measured with an enzyme method.

Coronary heart disease was diagnosed when patients had typical symptoms and the typical change in electrocardiogram, the abnormal image of coronary artery through coronary CT angiography or the definitely past illness history diagnosed by a cardiologist. Stroke was diagnosed in two ways: one was a patient with a self-reported medical history diagnosed by neurological physicians; another was a new stroke that happened when being hospitalized, which is very occasional. Diabetic Kidney Disease (DKD) was diagnosed by elevated urinary albumin excretion (urinary albumin/creatinine ratio ≥30 mg/g or 24-h urinary albumin ≥30 mg/24 h) and a reduced estimated glomerular filtration rate (eGFR) in the absence of other primary causes of kidney damage or CKD previously diagnosed. Those with the following symptoms should be considered as possibly having non-diabetic kidney disease and be referred to a nephrologist: abnormal urinary sediment (hematuria, proteinuria with hematuria, and tubular urine), rapid decline in eGFR, no retinopathy, rapid urine increase of UACR within a short period, or nephrotic syndrome. Diabetic retinopathy was diagnosed by an ophthalmologist based on funduscopy. Diabetic neuropathy was diagnosed with a 128 Hz tuning fork combined with a 10-g monofilament test.

### Diabetes Control Goals

The cardiovascular risk factor control goals were assessed according to the Chinese Diabetes Society Standards of Medical Care for Type 2 Diabetes in 2019 (6): HbA1c <7.0%, blood pressure <130/80 mmHg, total cholesterol <4.5 mmol/L, LDL cholesterol <2.6 mmol/L without and with cardiovascular disease <1.8 mmol/L, HDL cholesterol >1.0 mmol/L in men and >1.3 mmol/L in women, and triglycerides <1.7mmol/L. Meanwhile, we used the ADA Standards of Medical Care in Diabetes 2015 to assess cardiovascular disease risk factors control: HbA1c <7%, blood pressure <140/90 mmHg, LDL cholesterol <2.6 mmol/L (7).

### Statistical Analyses

The baseline characteristics were presented for continuous variables as means (standard deviation) or categorical variables as frequencies (percentages). The yearly percentages of patients’ attainment of the Chinese Diabetes Society or ADA goals for cardiovascular disease risk factor control were calculated using logistic regression adjusted for age and sex (except for sex-specific analyses). The linear trend across time was tested using attainment of goals as the outcome variable and year as a continuous variable in the model. P-values <0.05 were taken to indicate statistical significance. All statistical analyses were performed using IBM SPSS Statistics for Windows, version 25.0 (IBM Corp., Armonk, New York).

## Results

The present study included 3,245 inpatients with type 2 diabetes. General characteristics of the study population are shown in **Table 1**. The mean value of age was 59.2. Mean values of BMI, systolic/diastolic blood pressure, HbA1c, total cholesterol, LDL cholesterol, HDL cholesterol, and triglycerides were 26.5 kg/m^2^, 136/81 mmHg, 8.34%, 4.93 mmol/L, 2.95 mmol/L, 1.15 mmol/L (for men 1.06 mmol/L, and for women for 1.23 mmol/L), and 1.50 mmol/L, respectively. The proportions of medication use for blood pressure, blood glucose and lipid were 53.3, 96.5, and 44.3%, respectively.

**Table 1:** Baseline characteristics of inpatients with type 2 diabetes.

	Average	Men	Women	P value
No. of participants	3245	1545	1700	
Age, years	59.2 (12.1)	57.7 (11.9)	60.6 (12.1)	0.00
Body mass index, kg/m^2^	26.5 (4.15)	26.4 (3.67)	26.6 (4.54)	0.16
Blood pressure, mmHg				
Systolic	136 (19.1)	136 (18.5)	137 (19.6)	0.44
Diastolic	81.0 (11.0)	82.4 (11.2)	79.8 (10.7)	0.00
Total cholesterol, mmol/L	4.93 (1.46)	4.71 (1.39)	5.12 (1.50)	0.00
High-density lipoprotein cholesterol, mmol/L	1.15 (0.37)	1.06 (0.34)	1.23 (0.38)	0.00
Low-density lipoprotein cholesterol, mmol/L	2.95 (1.13)	2.81 (0.99)	3.08 (1.24)	0.00
Triglycerides, mmol/L	1.50 (1.20)	1.49 (1.26)	1.51 (1.16)	0.40
HbA1c, %	8.34 (2.00)	8.38 (2.01)	8.30 (1.99)	0.09
HBA1c, mmol/moL	67.6 (21.9)	68.1 (22.0)	67.2 (21.7)	0.93
Uses of medication, %				
Antihypertensive medication	53.3	51.0	55.3	0.02
Glucose-lowering medication	96.5	97.0	96.1	0.16
Oral hypoglycemic agents	33.4	32.9	33.9	0.55
Insulin	63.2	64.2	62.3	0.26
Lipid-lowering medication	44.3	43.7	44.8	0.52

Data are mean (SD) or percentage.

**Tables 2** and **3** showed the adjusted percentages of patients’ attainment of the Chinese Diabetes Society and ADA cardiovascular disease risk factor control goals from 2013 to 2017. Percentages of patients who achieved HbA1c <7% according to both Chinese Diabetes Society and ADA goals were 26.7, 24.6, 28.3, 30.7, and 30.5% for 2013–2017 (P for trend = 0.065), respectively. A notable increase in the percentages of patients achieving blood pressure <130/80 mmHg according to the Chinese Diabetes Society goal was observed between 2013 and 2017 as 14.8, 16.6, 18.1, 21.7, and 16.2% (P for trend = 0.011), respectively. While using ADA goals for blood pressure <140/90 mmHg, the proportions were 45.7, 47.7, 55.2, 50.6, and 48.4 from 2013 to 2017 (P for trend = 0.017), respectively. The percentages of achieving the Chinese Diabetes Society goal for LDL cholesterol control were lower [28.5, 28.0, 33.6, 36.4, and 33.2% from 2013 to 2017 (P for trend = 0.004), respectively] than the percentages of achieving the ADA goal for LDL cholesterol control [36.0, 36.0, 42.6, 42.7, 41.2% from 2013 to 2017 (P for trend = 0.014), respectively]. According to the Chinese Diabetes Society goal, we also calculated the total cholesterol control rates which were also improved through years (P for trend = 0.006). However, the trend of HDL and triglyceride control goals was decreasing through the years. We further evaluated the percentages of patients having a normal lipid profile (simultaneously achieving targets of total cholesterol, triglycerides, LDL cholesterol, and HDL cholesterol), and it had a declining trend from 10.5 in 2013 to 8.0% in 2017. Percentages of patients meeting all the targets like HbA1c, blood pressure, total cholesterol, triglycerides, LDL cholesterol, and HDL cholesterol in terms of the Chinese Diabetes Society targets increased from 0.2% in 2013 to 0.9% in 2017 but was not significant (P for trend =0.21). The percentages of patients who met all three ADA goals had significant increases which were 4.3, 5.8, 8.7, 8.0, and 9.0% from 2013 to 2017 (P for trend =0.004), respectively.

**Table 2:** Percentage of inpatients with type 2 diabetes attaining the Chinese Diabetes Society goals for Prevention and Treatment of Diabetes from 2013 to 2017.

	Year					P value
	2013	2014	2015	2016	2017	
No. of participants						
Men	259	281	275	346	384	
Women	348	321	264	393	374	
Total	607	602	539	739	758	
HbA1c <7.0%						
Men	26.2	24.9	29.9	24.2	29.9	0.299
Women	27.3	24.3	26.5	36.4	31.0	0.004
Average	26.7	24.6	28.3	30.7	30.5	0.065
Blood pressure <130/80 mmHg						
Men	14.3	14.6	13.1	19.1	14.8	0.270
Women	15.6	18.3	23.1	24.0	17.5	0.017
Average	14.8	16.6	18.1	21.7	16.3	0.011
Normal lipids						
Men	16.6	10.6	9.20	16.7	10.6	0.007
Women	5.20	4.70	8.30	7.90	5.60	0.192
Average	10.4	7.50	8.50	12.1	8.00	0.019
Triglyceride <1.7 mmol/L						
Men	61.0	56.4	61.6	61.7	54.0	0.120
Women	59.9	61.2	61.3	61.6	52.4	0.053
Average	60.3	59.0	61.4	61.7	53.3	0.006
Total cholesterol <4.5 mmol/L						
Men	43.6	46.5	54.1	49.9	43.4	0.038
Women	29.9	31.1	34.1	40.0	34.2	0.040
Average	36.4	38.5	43.8	44.7	38.6	0.006
Low-density lipoprotein cholesterol <2.6 mmol/L (without CHD)/<1.8 mmol/L (with CHD)						
Men	35.9	34.9	38.6	38.4	37.7	0.854
Women	22.3	21.7	29.2	34.5	28.9	<0.001
Average	28.5	28.0	33.6	36.4	33.2	0.004
High-density lipoprotein cholesterol >1.0 mmol/L (men)/>1.3 mmol/L (women)						
Men	62.9	47.3	39.1	44.5	50.2	<0.001
Women	39.1	32.1	36.4	35.9	29.4	0.061
Average	49.9	39.3	37.2	40.0	39.6	<0.001
All three						
Men	0.05	0.7	1.5	1.7	0.5	0.151
Women	0.3	0	0.4	0.5	1.3	0.149
Average	0.2	0.3	0.9	1.1	0.9	0.211

Values are adjusted for age and sex (total samples analyses only).

**Table 3:** Percentage of inpatients with type 2 diabetes attaining the American Diabetes Association goals of cardiovascular risk factors from 2013 to 2017.

	Year					P value
	2013	2014	2015	2016	2017	
No. of participants						
Men	259	281	275	346	384	
Women	348	321	264	393	374	
Total	607	602	539	739	758	
HbA1c <7.0%						
Men	26.2	24.9	29.9	24.2	29.9	0.299
Women	27.3	24.3	26.5	36.4	31.0	0.004
Average	26.7	24.6	28.3	30.7	30.6	0.065
Blood pressure <140/90 mmHg						
Men	41.7	49.8	54.5	50.6	50.0	0.056
Women	48.7	45.7	56.1	50.5	46.6	0.089
Average	45.7	47.7	55.2	50.6	48.4	0.017
Low-density lipoprotein cholesterol <2.6 mmol/L						
Men	41.3	40.5	47.1	45.0	45.0	0.471
Women	31.4	31.7	38.6	40.5	37.6	0.033
Average	36.0	36.0	42.6	42.7	41.2	0.014
All three						
Men	3.9	6.4	10.6	7.8	8.3	0.047
Women	4.6	5.3	6.8	8.2	9.5	0.058
Average	4.3	5.8	8.7	8.0	9.0	0.004

Values are adjusted for age and sex (total samples analyses only).

The prevalence of major diabetes complications including coronary heart disease (31.7 *vs*. 31.9%), stroke (16.7 *vs*. 14.8%), diabetic kidney disease (37.9 *vs*. 35.8%), diabetic retinopathy (48.0 *vs*. 46.5%, neuropathy (63.1 *vs*. 61.9%) and diabetic foot (0.8 *vs*. 1.2%) were stable from 2013 to 2017 (**Table 4**).

**Table 4:** Prevalence of major diabetic complications among inpatients with type 2 diabetes in Tianjin General Hospital from 2013 to 2017.

	Year					P value
	2013	2014	2015	2016	2017	
No. of participants						
Men	259	281	275	346	384	
Women	348	321	264	393	374	
Total	607	602	539	739	758	
Coronary heart disease						
Men	35.9	29.2	25.8	30.1	35.2	0.041
Women	28.1	27.1	29.2	22.1	29.0	0.167
Average	31.7	28.1	27.3	25.9	31.9	0.044
Stroke						
Men	18.9	16.7	16.4	15.0	13.8	0.491
Women	14.9	16.8	16.3	12.4	15.8	0.498
Average	16.7	16.8	16.3	13.7	14.8	0.420
Diabetic kidney disease						
Men	45.9	42.0	35.7	41.6	38.5	0.144
Women	31.3	35.9	39.4	30.0	33.5	0.098
Average	37.9	38.8	37.3	35.5	35.8	0.695
Diabetic retinopathy						
Men	50.6	50.9	50.2	45.4	46.6	0.509
Women	46.0	51.1	46.2	43.2	46.6	0.346
Average	48.0	51.0	48.2	44.3	46.5	0.161
Neuropathy						
Men	69.5	67.3	63.2	63.6	65.1	0.488
Women	57.7	59.9	57.2	58.9	59.2	0.960
Average	63.1	63.4	60.1	61.2	61.9	0.767
Diabetic foot						
Men	0	0.05	0	0.30	1.00	0.065
Women	1.40	0.60	1.10	0.80	1.30	0.796
Average	0.80	0.3	0.60	0.50	1.20	0.349

Values are adjusted for age and sex (total samples analyses only).

**Table 5** showed the adjusted prevalence of patients’ uses of different medications. Patients who used lipid-lowering agents were 44.3, 41.5, 39.8, 44.3, and 49.6% from 2013 to 2017 (P for trend = 0.004), respectively. A notable increase in the percentage of insulin usage was found with 62.2, 61.5, 60.0, 63.2, and 67.6% from 2013 to 2017 (P for trend = 0.046). **Table 6** showed prevalence of HbA1c below and above 7% among inpatients with type 2 diabetes attaining the Chinese Diabetes Society cardiovascular disease risk factor control goals from 2013 to 2017. Patients with normal lipids, triglyceride <1.7 mmol/L, or total cholesterol < 4.5 mmol/L were more likely to achieve the HbA1c <7% goal from 2013 to 2017 (all Ps for trend <0.05). Patients with blood pressure <130/80 mmHg, LDL cholesterol <2.6 mmol/L (without CHD)/< 1.8 mmol/L (with CHD), or HDL cholesterol >1.0 mmol/L (men)/> 1.3 mmol/L (women) were also more likely to achieve HbA1c <7% goal from 2013 to 2017, however, these trends were not significant (all P for trend >0.05).

**Table 5:** Percentage of uses of medication from 2013 to 2017 among inpatients with type 2 diabetes.

	Year					P value
	2013	2014	2015	2016	2017	
No. of participants						
Men	259	281	275	346	384	
Women	348	321	264	393	374	
Total	607	602	539	739	758	
Uses of antihypertensive medication						
Men	56.0	50.1	46.7	51.1	51.2	0.310
Women	57.9	57.0	47.6	56.0	56.0	0.080
Average	56.9	53.7	47.4	53.8	53.7	0.023
Uses of lipid-lowering medication						
Men	43.6	39.5	38.2	46.2	48.4	0.043
Women	44.9	43.5	41.2	42.5	50.9	0.088
Average	44.3	41.5	39.8	44.3	49.6	0.004
Uses of Glucose-lowering medication						
Men	96.9	97.1	96.8	96.5	97.6	0.924
Women	96.6	94.1	97.7	94.4	98.1	0.013
Average	96.8	95.5	97.2	95.4	97.9	0.050
Uses of oral hypoglycemic agents						
Men	33.6	34.2	40.4	30.1	28.6	0.020
Women	35.3	34.3	34.1	34.1	31.9	0.904
Average	34.5	34.2	37.3	32.2	30.3	0.086
Uses of insulin						
Men	63.3	63.0	56.8	66.5	69.0	0.021
Women	61.2	60.1	63.6	60.3	66.3	0.381
Average	62.2	61.5	60.0	63.2	67.6	0.046

All analyses adjusted for age and sex (total samples analyses only).

**Table 6:** Prevalence of HbA1c below and above 7% among inpatients with type 2 diabetes attaining the Chinese Diabetes Society goals for Prevention and Treatment of Diabetes from 2013 to 2017.

	Year					P value
	2013	2014	2015	2016	2017	
No. of participants						
HbA1c < 7%	163	148	152	227	231	
HbA1c ≥ 7%	444	454	387	512	527	
Total	607	602	539	739	758	
Blood pressure <130/80 mmHg						0.110
HbA1c < 7%	22.0	27.0	32.0	33.1	38.2	
HbA1c ≥ 7%	78.0	73.0	68.0	66.9	61.8	
Normal lipids						0.042
HbA1c < 7%	27.9	26.7	51.1	39.3	45.2	
HbA1c ≥ 7%	72.1	73.3	48.9	60.7	54.8	
Triglyceride <1.7 mmol/L						0.010
HbA1c < 7%	30.0	25.6	29.7	33.0	37.2	
HbA1c ≥ 7%	70.0	74.4	70.3	67.0	62.8	
Total cholesterol < 4.5 mmol/L						0.005
HbA1c < 7%	30.9	25.1	32.8	34.2	40.7	
HbA1c ≥ 7%	69.1	74.9	67.2	65.8	59.3	
Low-density lipoprotein cholesterol <2.6 mmol/L (without CHD)/< 1.8 mmol/L (with CHD)						0.112
HbA1c < 7%	28.2	28.0	35.5	36.9	37.0	
HbA1c ≥ 7%	71.8	72.0	64.5	63.1	63.0	
High-density lipoprotein cholesterol >1.0 mmol/L (men)/>1.3 mmol/L (women)						0.078
HbA1c < 7%	27.1	24.6	33.5	33.6	32.7	
HbA1c ≥ 7%	72.9	75.4	66.5	66.4	67.3	

All analyses adjusted for age and sex.

## Discussion

The present study found that control rates of major cardiovascular risk factors including HbA1c, blood pressure, and LDL cholesterol according to both Chinese Diabetes Society and ADA goals were improved from 2013 to 2017 among hospitalized patients in China. However, there still exists a gap between the guidelines and practice.

It is well known that patients with type 2 diabetes who have poor control of glycemia, high blood pressure, and dyslipidemia are at a higher vascular damage risk for the development of microvascular and macrovascular complications, which will lead to the worse prognosis and quality of life and bring a great burden for medical costs as well (12, 13). Some studies have found that complications of diabetes can be reduced by optimal management of blood glucose, blood pressure, and blood lipid (14). Thus several international and national associations such as ADA and the Chinese Diabetes Society recommend the simultaneous control of HbA1c, blood pressure, and LDL cholesterol, which was referred to the “ABC targets of diabetes”, but the control of the risk factors was suboptimal (15, 16).

In the US National Health and Nutrition Examination Survey in 2003–2006, 58.2% of patients with type 2 diabetes had an HbA1c <7%, 44.2% of patients were at a blood pressure goal of <130/80mmHg, 43.2% of patients had an LDL cholesterol<2.6 mmol/L, and 10.2% of patients were simultaneously at HbA1c, blood pressure and LDL cholesterol control goals, while those on insulin treatment were only 5.4% to get three goals (17). A multicenter observational study from China referred to as the “3B study” reported that 47.7%, 28.4%, and 36.1% of patients with type 2 diabetes achieved the individual target goal for control of blood glucose (HbA1c <7%), blood pressure (<130/80 mmHg), and blood lipids (total cholesterol <4.5 mmol/L), respectively, and only 5.6% of patients achieved all 3 target goals (18). A population-based retrospective cohort study conducted on 144,271 primary care patients with type 2 diabetes in Hong Kong from 2008 to 2011 has indicated that 47%, 37%, and 45% of patients with diabetes met the target levels for HbA1c, blood pressure, and LDL cholesterol, and only one tenth of the patients achieved all three goals and the reduction of CVD was related to the number of the increased targets attainment (5). However, all these studies were from outpatients. Comparing with the previous studies, the percentages of the inpatients attaining the goals of HbA1c <7% and blood pressure <130/80 mm Hg in the present study increased from 25.9% and 16.4% in 2013 to 28.8% and 20.8% in 2017, respectively. However, the control rates in the current study were still lower compared with previous studies mentioned above. An important difference was that the previous studies focused on the outpatients from clinic or community hospitals, however, the present study assessed the hospitalized patients.

Although the attaining target rates were suboptimal in the present study, the trend of the patients achieving all three ADA goals including HbA1c<7%, blood pressure <140/90 mmHg and LDL cholesterol <2.6 mmol/L was significantly improved from 2013 to 2017. The proportion of the patients achieving all these three goals was rising up from 4.5% in 2013 to 9.1% in 2017. Meanwhile, we evaluated the proportion of achieving blood glucose, blood pressure and all types of lipid (including triglycerides, total cholesterol, LDL cholesterol, and HDL cholesterol) targets based on the Chinese Diabetes Society goals and found that rates for all indices simultaneously standardized were still very low (less than 1%) from 2013 to 2017. Reasons for the improved proportion are speculated as follows: First, the diabetes management mainly reinforced the community-based interventions and focused on the patients education, team management and self-management support, which will help to improve the treatment effect of diabetes (19–22). Second, as the social-economic level improved, diabetes patients concerned more about the health status and pursued longevity and good life quality. Thus they will strengthen the self-management which will improve the attainment of metabolic indices to some extent. Third, the increasing medication usage was beneficial to the improvement of attaining ADA control goals. As shown, the usage rates for lipid-lowering treatment were significantly improved from 2013 to 2017, which may be the major cause for the improved trend of attaining lipid control goal. Moreover, the increased insulin usage rates may result in the improved control goal of HbA1c. Over the years, several new classes of antihyperglycemic agents, including GLP-1 receptor agonists and sodium–glucose cotransporter inhibitors 2 (SGLT-2) are widely used in the type 2 diabetes therapy, which have extra benefits for weight loss and blood-pressure reductions, even reducing the risk of major cardiovascular adverse events, renal events, and hospitalization rates for heart failure (23–26). In the future study, we will focus on the impact of these new hypoglycemic agents on the control of cardiovascular risk factors.

The present study found that the major complications of diabetes did not change from 2013 to 2017. The prevalence of complications of diabetes in the present study was higher than the “3B STUDY” in China (18) and the “DISCOVER STUDY” (27) around the world. The major reason was that the participants in our study were hospitalized patients who had more serious conditions, comparing with the patients in the above studies, which were mainly outpatients or from the primary healthcare settings. Thus the intensive controls of glycemia, blood pressure and lipids mainly decreased the microvascular and macrovascular complications among patients with type 2 diabetes (5, 28–30). Furthermore, as reported in previous literatures, the increased utilization of insulin in patients with type 2 diabetes was usually associated with poor glycemic control in the long run. The gradual improvement in glycemic control (HbA1c less than 7) over the same period was mainly seen in women as shown in **Tables 2** and **3**, but not men or the whole population sample (this is not significant), which may explain the lack of improvement of diabetic complications over the same period.

There are several strengths of our study. First, previous studies mainly enrolled the outpatients from the clinic, primary healthcare agents or the health survey who are in a relative better health status and compliance. The present study selected the inpatients from a tertiary comprehensive hospital, which targeted to the patients who had a worse health status and a more complicated condition. Second, we observed the metabolic indices for five consecutive years that were a long term in the real-world clinical settings and the key indicators had no missing. Third, we not only used the Chinese Diabetes Society standards, but also the ADA goals to assess the attainments of the targets, which avoided the guideline discrepancies leading to the different results. Several limitations should be addressed. First, this was a cross-sectional study that had a deficiency for causal inferences especially between the predictive risk factors and the outcomes. Second, this was a single-center study lack of representativeness for the general situations of inpatients in all tertiary hospitals. Third, some data like coronary heart disease and stroke were collected from the patients’ self-reported medical history, which might result in the recalling bias.

In conclusion, the present study found that there was an improving trend of the control rates of blood glucose, blood pressure and blood lipid from 2013 to 2017 among inpatients with type 2 diabetes in Chinese. There is still a big gap between the rates of cardiovascular risk factors and guidelines, especially for the inpatients with type 2 diabetes. Thus there are more efforts to do in the clinical work to help type 2 diabetes patients get better management for the comorbidities and complications.

## Data Availability Statement

The raw data supporting the conclusions of this article will be made available by the authors, without undue reservation.

## Ethics Statement

The study and analysis plan were approved by the Ethical Committee of Tianjin Medical University General Hospital. We did not obtain written informed consent in the present study because we used anonymized data compiled from electronic medical records.

## Author Contributions

YF, LD, and RW researched data. XY, QL, and GH wrote the manuscript. GH and ML reviewed and revised the manuscript. ML and GH are the guarantor of this work and, as such, had full access to all the data in the study and take responsibility for the integrity of the data and the accuracy of the data analysis. All authors contributed to the article and approved the submitted version.

## Funding

This work was supported by research grants from the National Natural Science Foundation of China 81830025, 81620108004 and 81570699, and National Key R&D Program of China 2019YFA0802502. We acknowledge the supports of the Tianjin Municipal Science and Technology Commission 17ZXMFSY00150.

## Conflict of Interest

The authors declare that the research was conducted in the absence of any commercial or financial relationships that could be construed as a potential conflict of interest.
